# Peer Stress Spills Over to Family Stress in the Context of Emotion Regulation Difficulties: A Daily Diary Study with Chinese Adolescents

**DOI:** 10.1007/s10964-024-01962-3

**Published:** 2024-03-11

**Authors:** Hui Wang, Yutong Zhang, Molly Elizabeth Hale, Sihan Liu, Jianjie Xu, Chenxi Zhu, Cynthia Suveg, Zhuo Rachel Han

**Affiliations:** 1 Department of Psychology, Faculty of Arts and Sciences, Beijing Normal University at Zhuhai, Zhuhai, China; 2 Faculty of Psychology, Beijing Normal University, Beijing, China; 3 Department of Population Health Sciences, Weill Cornell Medicine, New York, NY USA; 4 Department of Psychology, University of Georgia, Athens, GA USA; 5 Division of Psychology and Language Sciences, University College London, London, UK

**Keywords:** Peer stress, Family stress, Daily diary, Emotion regulation, Adolescence

## Abstract

Conflict in peer and family relationships becomes more common in the adolescent period when compared to previous developmental periods. These typical developmental challenges can be exacerbated in the context of poor emotion regulation skills. Using daily diary data, the current study examined the stress spillover effects of peer and family stress on one another, as well as the moderating role of emotion regulation challenges (i.e., emotional inhibition, dysregulation). A sample of 310 Chinese adolescents (*M*_*age*_ = 13.02 years, *SD* = 0.76 years, 50.7% boys) completed an initial measure of emotion regulation difficulties, then reported on peer and family stress for 10 consecutive weekdays. Results indicated that there was an overall same-day peer stress spillover effect in which adolescents’ peer stress on a given day was negatively associated with later conflictual interactions with their parents. Further, the relation between peer stress and same- and next-day family stress was exacerbated in the context of high levels of emotional inhibition. Family stress did not significantly relate to next-day peer stress, nor was this association moderated by difficulties with emotion regulation. These results highlight the temporal sequence of daily peer-to-family stress spillover. Though emotional inhibition may be culturally adaptive for maintaining interpersonal harmony, it can be maladaptive in managing stress for Chinese adolescents.

## Introduction

Adolescents often face relational stress in their daily lives, primarily stemming from school and family—two critical developmental contexts (Mayfield & Fosco, 2021). Relational stress associated with peers and family may have immediate impacts on current experiences but can also carry over from school to home, impacting subsequent interactions with parents and, in turn, influencing peer relations the following day (Bai et al., 2017). This phenomenon, known as the school-to-home spillover (Lehman & Repetti, 2007), has been shown to have reciprocal associations between peer and family stress at the daily level (Chung et al., 2011; Timmons & Margolin, 2015). However, findings are largely based on daily diary methods that capture peer- and family-related stress simultaneously each day, which hinders the ability to establish temporal precedence of spillover effects. Moreover, limited research has explored how differences in adolescents’ emotion regulation difficulties may either buffer or exacerbate the stress spillover process. To address these gaps, this study assessed peer and family stress at multiple time points throughout the day and examined the temporal sequence of the stress spillover process within school and family contexts in a sample of Chinese adolescents. Additionally, this study investigated the moderating role of adolescent emotion regulation difficulties in this stress spillover process.

### Peer and Family Stress in Daily Lives

As youth transition to middle school, they are exposed to significant changes in school and familial environments, resulting in higher reports of perceived stress when compared to earlier in childhood (Bai & Repetti, 2018). This increase in stress is likely due to changes in adolescents’ social landscape such that they are attempting to manage parental expectations while navigating emotionally-intense friendships (Kiang & Buchanan, 2014). The transition to middle school is met with larger class sizes, rotating class schedules, and changing peer groups (Erath et al., 2019; Madjar & Cohen-Malayev, 2016). In the midst of these social changes, parent-child relationships also undergo significant transformations with adolescents seeking greater autonomy, resulting in more conflictual interactions with parents than previously in childhood (Silva et al., 2020). Despite increased levels of peer and familial stress during adolescence, studies have yet to fully examine the temporal effects of how one domain of stress may influence, or “spillover,” into another.

Like all youth, the transition to adolescence for Chinese youth is marked by the increased importance of peer relationships, making adolescents especially vulnerable to social stressors (Brown & Larson, 2009; Ladd & Troop-Gordon, 2003). Chinese culture emphasizes interpersonal harmony (Oyserman et al., 2002), a value that challenges individuals to be sensitive to peer feedback and social evaluation as a way of shaping culturally-appropriate behaviors and adapting to social norms (Chen, 2012; Chen et al., 2018). When compared to White adolescents, Chinese youth spend a greater portion of their day in the school setting, with classes beginning before 8 AM and letting out after 5 PM. These longer school days offer more opportunities for interactions, but they also provide greater chances for interpersonal tension with peers (Xu & Minca, 2008). Given the unique cultural context in which Chinese adolescents develop, it is vital to examine the spillover process of peer stress into family functioning to understand these social contexts better.

The Stress Spillover Model (Almeida et al., 1999) proposes that when an individual experiences stress in one domain, it may impact stress in another context. In examining the school-to-home spillover process, the bioecological theory offers a robust theoretical framework for understanding how different elements within the microsystem (e.g., peer and family stress), can influence each other (Bronfenbrenner & Ceci, 1994). The microsystem encompasses the immediate environments in which an individual actively participates, and these environments are interconnected in such a way that experiences in one context (e.g., school) can influence interactions and experiences in another context (e.g., home). This interconnection suggests that peer stress, characterized by challenges such as arguing with friends, bullying, or social exclusion, can transcend the school environment and affect family interactions. Specifically, peer stress at school on a given day can lead to emotional distress, behavioral changes, and academic issues in adolescents, which, in turn, strain family dynamics later at home as parents react to and cope with the adolescent’s altered behavior and emotional states (Flook & Fuligni, 2008; Mayfield & Fosco, 2021).

The interrelatedness of peer stress and family dynamics has received considerable empirical support. For instance, some studies have identified daily spillover effects from school to home. Specifically, fifth-grade children have reported increased negative interactions with parents on days following higher peer problems at school (Lehman & Repetti, 2007). Additionally, stressful interpersonal experiences at school have been shown to influence adolescents’ subsequent views on family dynamics, leading to perceptions of reduced warmth and increased conflict with parents over an 8-week period (Bai et al., 2017).

Bioecological theory (Bronfenbrenner & Ceci, 1994) also supports the idea that interactions perceived as stressful in the home can impact peer relations. For example, children from unsupportive family environments tend to have difficulties in forming positive peer relationships (Franz & Gross, 2001). Conflict with mothers has also been linked to greater peer rejection among adolescents (Liu et al., 2020), whereas parental rejection is associated with increased peer victimization (Kaufman et al., 2020). Furthermore, research has documented a bi-directional relationship between peer problems and parent-adolescent relationships, both concurrently and with a lag of 1 or 2 days (Chung et al., 2011; Timmons & Margolin, 2015).

Though informative, much prior work has involved youth completing daily diaries about peer- and family-related stress at the same time each day (e.g., Lehman & Repetti, 2007; Timmons & Margolin, 2015), which hinders the ability to determine the temporal precedence of spillover effects. Assessing temporal precedence in stress spillover is critical given that reported stress in one domain (e.g., peers) may be artificially inflated by state-dependent recall (e.g., in light of a recent conflictual interaction with parents; Bai et al., 2017). By assessing peer and family stress at different time points throughout the day, the current study will more clearly delineate the temporal precedence of how stress in one social context may impact another in a sample of Chinese adolescents.

### Emotion Regulation Difficulties as a Moderator of Daily Stress Spillover

Converging evidence has documented the effect of daily stress spillover in general; however, individual-level variables, such as emotion regulation, may influence these spillover effects. In particular, difficulties with expression, maintenance, and modification of emotional experiences across various social contexts, can impact the stress spillover process (Cole & Hall, 2008). Stress and coping theory suggests that the emotion regulation strategies adolescents employ to cope with stress can influence their reactions to interpersonal stress (e.g., peer stress) and the transmission of that stress (Lazarus & Folkman, 1984). Previous studies have highlighted two maladaptive emotion regulation strategies: emotional inhibition and dysregulation (Richardson, 2017; Rothenberg et al., 2019). Emotional inhibition refers to chronic attempts to mask emotional experiences, avoiding outward displays (e.g., hiding feelings of sadness; Gross & John, 2003). In contrast, dysregulation encompasses patterns of uncontrolled and excessive emotional displays in socially inappropriate ways, such as slamming doors and screaming when angry (Zeman et al., 2001, 2010).

Research indicates that the use of emotional inhibition and dysregulation exacerbate the effects of daily interpersonal stress on daily emotional well-being. For instance, daily stress exhibited a stronger negative association with daily positive affect in the context of high levels of adolescent emotional inhibition than at low levels (Richardson, 2017). Another study showed that sadness dysregulation enhanced the daily spillover effect of adolescents’ sadness on their depressive symptoms (Rothenberg et al., 2019). It is also possible that emotional inhibition might lead to an accumulation of unresolved stress, thereby intensifying the impact of daily stressors (Gross, 2014). When adolescents engage in emotional inhibition, they are more likely to experience a build-up of negative emotions, which can spillover and amplify their reactions to stress within both peer and family contexts (Aldao et al., 2010). Conversely, dysregulated expression can disrupt family dynamics and peer relationships, escalating every day conflicts and stress (Timmons & Margolin, 2015). These emotion regulation difficulties not only heighten adolescents’ stress responses but also have the potential to trigger a reciprocal increase in stress within their social environments, thereby creating a feedback loop that perpetuates and magnifies the stress spillover process.

### Chinese Culture and Attitudes Towards Emotion Regulation

Studies have underscored the value of culture in shaping individuals’ beliefs and expectations regarding emotional expression. In Chinese culture, to maintain interpersonal harmony, individuals are presumed to minimize emotional expression to foster greater social cohesion (Deng et al., 2019). This cultural norm likely encourages Chinese adolescents to employ emotional inhibition as a means to navigate interpersonal conflicts (Wei et al., 2013). However, this minimization of emotional expression for the betterment of group cohesion may come at the cost of adolescents’ psychological well-being, such that greater emotional inhibition may result in prolonged experiences of negative emotions (Zeman et al., 2001, 2010). Conversely, dysregulation of negative emotions is typically viewed as inappropriate and unexpected within the Chinese cultural context, making Chinese adolescents less likely to adopt this approach, as it could disrupt social harmony (Deng et al., 2019).

A cultural lens is also critical to understanding the relations between inhibition and dysregulation. Studies in Western populations suggest that emotional inhibition can lead to a build-up of unexpressed emotions, eventually resulting in dysregulated outbursts (Gross & Cassidy, 2019). However, in collectivist societies like China, where emotional restraint is often socially reinforced, emotional inhibition might not necessarily lead to dysregulation; instead, they may function as distinct coping mechanisms, each serving unique psychological and social functions (Chen & Danish, 2010; Wei et al., 2013). Research is needed to better understand the interplay between cultural factors and emotion regulation among Chinese adolescents, particularly in relation to the stress spillover process.

## Current Study

Although previous research provides initial evidence on the daily stress spillover within school and family settings, these studies typically measure daily peer and family stress at the same time point. This oversight hinders insights into how stress transmits sequentially across various social contexts. Moreover, while the significance of emotion regulation difficulties in the spillover process is recognized, the focus has predominantly been on Western adolescents, overlooking the potential influence of culture on emotion regulation and the stress spillover process in non-Western adolescents. To address these gaps, this study examines stress spillover in adolescence by: (1) assessing peer and family stress at multiple time points throughout the day to document the temporal sequence of the stress spillover process; (2) examining emotion regulation difficulties as moderators of the stress spillover process; (3) applying a cultural lens to the study of the stress spillover process in a sample of Chinese adolescents. The following hypotheses are put forth: (1) elevated peer stress at school is likely to lead to increased family stress at home, both on the same day and the following day (Hypothesis 1); (2) stress in the family context will spilloever to the peer domain the next-day (Hypothesis 2); and (3) both same-day and next-day stress spillover effects will be greatest the in the context emotion regulation difficulties (i.e., greater inhibition and dysregulation; Hypothesis 3).

## Method

### Participants

A total of 315 seventh-grade adolescents between 11- and 15-years-old (*M*_age_ = 13.05 years, *SD* = 0.77 years; 48.3% girls) participated in the present study. Participants were recruited from a public middle school in northeast China. All seventh-grade students were invited to participate. Adolescents who responded to less than 90% of the questionnaires or missed more than 3 days of daily diary entries were excluded from analyses (*n* = 5). The retained sample consisted of 310 adolescents (*M*_*age*_ = 13.02 years, *SD* = 0.76 years; 49.2% girls). In the final sample, 91.3% of the participants identified as Chinese Han ethnicity. The rest of participants reported their ethnicity as Manchu (2.5%), Hui (0.3%), Mongol (0.3%), and Korean (0.3%). Regarding parent education, the majority of the sample completed middle school (42.7%), with others reporting having completed college (25.8%), high school (24.7%), and less than middle school education (6.8%). The majority of families (76.9%) reported an annual household income below 72,000 RMB (approximately 10,000 USD), which was below the average family income of the city (77,363 RMB or approximately 10,750 USD; Heilongjiang Bureau of Statistics, 2020).

### Procedure

All study procedures were approved by the university’s institutional review board (IRB). Adolescents provided written assent in person prior to participating in this study at school. Meanwhile, parents signed online informed consent for their children’s participation. Following study enrollment, adolescents completed an initial questionnaire regarding emotion regulation difficulties, and parents reported on family demographics. Both sets of surveys were answered using Qualtrics. After baseline measures were completed, adolescents filled out daily diary entries regarding peer and family stress for 10 consecutive weekdays (i.e., Monday through Friday for two consecutive weeks). On each of the 10 weekdays, adolescents completed paper-and-pencil diary measures twice per day: at the end of the school day for peer stress at 5 PM (i.e., following the final class of the day) and at bedtime for family stress at approximately 9 PM. All of the bedtime diaries were brought to school the following morning. Research assistants visited the school every day, providing detailed instructions to adolescents about the daily procedures. They ensured that all instructions and materials were thoroughly understood and collected the daily diary entries concurrently. Of the 10 diary entries assigned for each form of stress, participants completed an average of 9.65 (*SD* = 0.82) diaries for peer stress and 9.16 (*SD* = 1.40) diaries for family stress.

### Measures

#### Adolescent Emotion Regulation Difficulties

The *Children’s Emotion Management Scales* (CEMS; Zeman et al., 2001, 2010) were used to assess adolescents’ emotion regulation. The CEMS consists of 36 items that assess adolescents’ ability to regulate anger, sadness, and worry. Adolescents indicate the frequency with which they employ three emotion management strategies for each emotion: (a) Inhibition—the tendency to suppress their emotional expression (e.g., “I’m afraid to show my sadness”); (b) Dysregulation—the use of socially inappropriate emotional expression (e.g., “I do things like slamming doors when I’m mad”); and (c) Emotion Regulation Coping—constructive control over their emotional behaviors (e.g., “I try to calmly deal with what is making me sad”). Items were rated on a 3-point scale ranging from 1 = “*hardly ever*” to 3 = “*often*.” Given the present study’s particular interest in emotion regulation difficulties, only the inhibition and dysregulation subscales were used. A composite score for each emotion regulation strategy was formed by averaging the items across the three emotions (i.e., dysregulation and inhibition items across anger, sadness, and worry were combined into two overall dysregulation and inhibition scores, respectively). Both the original English version of the CEMS (Zemen et al., 2001, 2010) and the Chinese version (Suveg et al., 2014) have demonstrated sound reliability and validity. Further, the measure has shown sound psychometric properties with adolescent samples (Cui et al., 2014; Sim & Zeman, 2005). In the current study, the internal consistency was adequate (α = 0.79 and 0.75 for inhibition and dysregulation, respectively).

#### Daily Peer Stress

For each of the 10 days of daily diary entries, adolescents were asked to rate to what extent they experienced peer stress during the school day. Peer stress was measured by four items (i.e., “Argued with a close friend,” “Had a lot of demands made by friends,” “Another schoolmate teased me,” and “I felt that my friends didn’t want to be around me.”). Participants responded to these items using a 4-point scale ranging from 1 = “*definitely false*” to 4 = “*definitely true*.” Items were aggregated to generate a total score for peer stress each day, with higher scores indicating more stress.This scale has exhibited adequate psychometric properties for application with adolescents (Bai & Repetti, 2018; Kiang & Buchanan, 2014), including studies focusing specifically on Chinese adolescents specifically (Flook, 2011; Xu et al., 2023). In this study, the internal consistency of the peer stress scale was satisfactory, with an average α of 0.87 across the 10-day period (α’s ranged from 0.74 to 0.95).

#### Daily Family Stress

In the same set of daily diary entries, adolescents were also asked to assess the level of family stress they experienced in the evening before bed. Family stress was evaluated using five items (i.e., “Punished or disciplined by parents,” “Argued with your mother about something,” “Argued with your father about something,” “Argued with another family member about something,” and “Had a lot of demands made by family.”) Like the peer stress scale, responses were given on a 4-point scale ranging from 1 = “definitely false” to 4 = “definitely true.” These responses were then combined to form a total score for family stress each day, with higher scores indicating greater stress. In the present study, the average α for family stress was 0.87 (α’s ranged from 0.81 to 0.90).

#### Sociodemographic Variables

Family demographic variables were assessed as potential control variables. Adolescents and their parents separately provided information on their age and gender (0 = “male,” 1 = “female”). Moreover, parents indicated their education level (1 = “primary school,” 2 = “secondary school,” and 3 = “university level”), monthly household income (ranging from 1 = “less than 2000 RMB” to 10 = “more than 20,000 RMB”), and ethnicity (1 = “Han,” 2 = “Manchu,” 3 = “Hui,” 4 = “Mongol,” and 5 = “Korean”).

### Analytic Plan

Multilevel modeling (MLM) was conducted using Hierarchical Linear Modeling 8 (HLM; Raudenbush et al., 2019) to test same- and next-day associations between peer and family stress, as well as the moderating effect of adolescent emotion regulation difficulties. Multilevel modeling, which allows for the simultaneous estimation of both between- and within-person effects, was used given the nested structure of these data (i.e., daily measures of peer and family stress nested within adolescents). Additionally, multilevel modeling in HLM handles missing data using full information maximum likelihood (FIML) estimation, compensating for when adolescents provide an unequal number of daily diary entries.

The potential control variables (i.e., adolescent age and gender, parental age and gender, parent educational level, household income, and ethnicity) were entered as covariates at Level 2 in all models. None of the variables were significantly related to same- or next-day associations and thus were dropped from the final models for the sake of parsimony.

#### Model 1: Same-Day Peer-to-Family Stress Spillover

To examine the temporal precedence of same-day associations between peer and family stress, adolescents’ daily levels of family stress served as the outcome variable, and peer stress was entered as the predictor. At level 1, the family stress for the *i*th adolescent on a given day *t* (*Family Stress*_*it*_) is represented as a function of the average level of daily family stress across the 10-day period (*β*_*0i*_) and same-day peer stress (person-centered) (*β*_*1i*_). The week (1 = “1st week,” 2 = “2nd week”; *β*_*2i*_) and day of the week (from 1 = “Mondy” to 5 = “Friday”; *β*_*3i*_) was entered as covariates to control for systematic changes in daily family stress over the 10-day period.

To test whether the within-person association between daily peer and family stress was moderated by adolescent emotion regulation, adolescents’ grand-mean centered scores for emotional inhibition and dysregulation were incorporated separately into Level 2 of the model as predictors of individual’s average levels of daily family stress (*γ*_*02*_) and the within-person association between peer stress and same-day family stress (*γ*_*11*_). Moreover, adolescents’ average peer stress (*γ*_*01*_) was grand-mean centered and entered as a Level 2 predictor of the intercept to control for between-person differences in average levels of daily peer stress. Equations for Levels 1 and 2 of the model are below.

Level 1:

$$\begin{array}{ll}{{Family}\,{Stress}}_{{it}}=&{\beta }_{0i}+{\beta }_{1i}\,{{Peer}\,{Stress}}_{{it}}+{\beta }_{2i}\,{{Week}}_{{it}}\\&+\,{\beta }_{3i}\,{Day}\,{of}\,{the}\,{{Week}}_{{it}}+{e}_{{it}}\end{array}$$

Level 2:

$$\begin{array}{ll}{\beta }_{0i}=&{\gamma }_{00}+{\gamma }_{01}\,{Average}\,{Peer}\,{{Stress}}_{i}\\&+\,{\gamma }_{02}\,{Inhibition}/{{Dysregulation}}_{i}+{u}_{0i}\end{array}$$

$${\beta }_{1i}={\gamma }_{10}+{\gamma }_{11}\,{Inhibition}/{{Dysregulation}}_{i}+{u}_{1i}$$

$${\beta }_{2i}={\gamma }_{20}+{u}_{2i}$$

$${\beta }_{3i}={\gamma }_{30}+{u}_{3i}$$

#### Model 2: Next-Day (Lagged) Peer-to-Family Stress Spillover

To examine next-day associations between peer and family stress, a similar HLM model to that of Model 1 was run; however, in Model 2, daily peer stress served as a predictor of next-day family stress after controlling for same-day family stress. At Level 1, adolescents’ next-day family stress was modeled as a function of the intercept (*β*_*0i*_), representing the individual’s average level of daily family stress across the 10-day period. The coefficient *β*_*1i*_ tested the effect of same-day peer stress (person-centered) on levels of next-day’s family stress. The week (*β*_*2i*_), day of the week (*β*_*3i*_), and same-day family stress (*β*_*4i*_) were included as control variables. At level 2, adolescent average peer stress (*γ*_*01*_) was grand-mean centered and controlled in the Level 1 intercept. Moreover, emotional inhibition and dysregulation were added into Level 2 of the model as predictors of individual’s average levels of daily family stress (*γ*_*02*_) and the within-person association between peer stress and next-day family stress (*γ*_*11*_).

Level 1:

$$\begin{array}{ll}{{Family}\,{Stress}}_{i(t+1)}=&{\beta }_{0i}+{\beta }_{1i}\,{Peer}\,{{Stress}}_{{it}}+{\beta }_{2i}\,{{Week}}_{{it}}\\&+\,{\beta }_{3i}\,{Day}\,{of}\,{the}\,{{Week}}_{{it}}+{\beta }_{4i}\,{Family}\,{{Stress}}_{{it}}+{e}_{{it}}\end{array}$$

Level 2:

$$\begin{array}{ll}{\beta }_{0i}=&{\gamma }_{00}+{\gamma }_{01}\,{Average}\,{Peer}\,{Stress}\\&+\,{\gamma }_{02}\,{Inhibition}/{{Dysregulation}}_{i}+{u}_{0i}\end{array}$$

$${\beta }_{1i}={\gamma }_{10}+{\gamma }_{11}\,{Inhibition}/{{Dysregulation}}_{i}+{u}_{1i}$$

$${\beta }_{2i}={\gamma }_{20}+{u}_{2i}$$

$${\beta }_{3i}={\gamma }_{30}+{u}_{3i}$$

$${\boldsymbol{\beta }_{4i}={\gamma }_{40}+{u}_{4i}}$$

#### Model 3: Next-Day (Lagged) Family-to-Peer Stress Spillover

At Level 1, next-day peer stress was modeled as the average level of peer stress across the 10-day period (*β*_*0i*_) and same-day family stress (person-centered) (*β*_*1i*_). The week (*β*_*2i*_), day of the week (*β*_*3i*_), and same-day peer stress (*β*_*4i*_) were included as Level 1 covariates. At level 2, emotional inhibition and dysregulation were added as predictors of individual’s average levels of daily peer stress (*γ*_*02*_) and the within-person association between family stress and next-day peer stress (*γ*_*11*_). Average family stress (*γ*_*01*_) was also included as a Level 2 covariate.

Level 1:

$$\begin{array}{ll}{{Peer}\,{Stress}}_{i(t+1)}=&{\beta }_{0i}+{\beta }_{1i}\,{Family}\,{{Stress}}_{{it}}+{\beta }_{2i}\,{{Week}}_{{it}}\\&+\,{\beta }_{3i}\,{Day}\,{of}\,{the}\,{{Week}}_{{it}}+{\beta }_{4i}\,{{Peer}\,{Stress}}_{{it}}+{e}_{{it}}\end{array}$$

Level 2:

$$\begin{array}{ll}{\beta }_{0i}=&{\gamma }_{00}+{\gamma }_{01}\,{Average}\,{Family}\,{Stress}\\&+\,{\gamma }_{02}\,{Inhibition}/{{Dysregulation}}_{i}+{u}_{0i}\end{array}$$

$${\beta }_{1i}={\gamma }_{10}+{\gamma }_{11}\,{Inhibition}/{{Dysregulation}}_{i}+{u}_{1i}$$

$${\beta }_{2i}={\gamma }_{20}+{u}_{2i}$$

$${\beta }_{3i}={\gamma }_{30}+{u}_{3i}$$

$${\beta }_{4i}={\gamma }_{40}+{u}_{4i}$$

## Results

### Preliminary Analyses

Table 1 shows the means, standard deviations, correlations, and gender differences among demographic and study variables. Independent sample *t*-tests revealed no adolescent gender differences in any of the study variables. Intraclass correlation analyses indicated that daily peer stress was positively associated with daily family stress (*r* = 0.06, *p* = 0.001). Moreover, adolescent dysregulation was positively related to the average levels of peer stress (*r* = 0.34, *p* < 0.001) and family stress (*r* = 0.22, *p* < 0.001).

**Table 1:** Means, Standard Deviations, and Correlation Coefficients among Study Variables

	Boys		Girls			*n* missing										
Variable	*M*	*SD*	*M*	*SD*	*t*-value	(%)	1	2	3	4	5	6	7	8	9	10
1. Peer stress	4.78	1.51	4.67	1.43	0.62	8.73	—									
2. Family stress	7.56	2.99	7.18	2.55	1.19	3.87	0.40^***^	—								
3. Inhibition	1.87	0.37	1.91	0.44	−0.69	3.23	−0.04	−0.02	—							
4. Dysregulation	1.57	0.40	1.57	0.40	−0.12	3.23	0.34^***^	0.22^***^	0.01	—						
5. C-age	13.08	0.74	12.95	0.77	1.58	0.01	0.07	−0.04	−0.05	−0.03	—					
6. C-gender	—	—	—	—	—	0.01	−0.04	−0.07	0.04	0.01	−0.09	—				
7. P-age	40.24	5.62	40.65	5.94	−0.63	5.14	−0.01	0.01	0.04	0.01	−0.03	0.04	—			
8. P-gender	0.70	0.45	0.78	0.40	—	5.14	0.07	0.03	−0.01	−0.01	−0.05	0.10	−0.20^***^	—		
9. P-education	2.23	0.51	2.15	0.57	1.37	5.14	−0.03	0.06	−0.05	−0.05	−0.14^*^	−0.08	0.01	−0.01	—	
10. Income	3.89	2.17	3.88	2.37	0.02	5.14	−0.07	−0.01	−0.05	−0.03	−0.17^**^	−0.01	−0.07	−0.03	0.31^***^	—
11. Ethnicity	1.07	0.33	1.05	0.35	0.43	5.14	−0.03	−0.02	0.01	0.02	0.12^*^	−0.03	−0.06	−0.10	−0.01	0.01

*C* child, *P* parent, *Peer Stress* average peer stress across 10 days, *Family Stress* average family stress across 10 days

* *p* < 0.05, ** *p* < 0.01, *** *p* < 0.001

### Model 1: Same-Day Peer-to-Family Stress Spillover

**Fig. 1:**
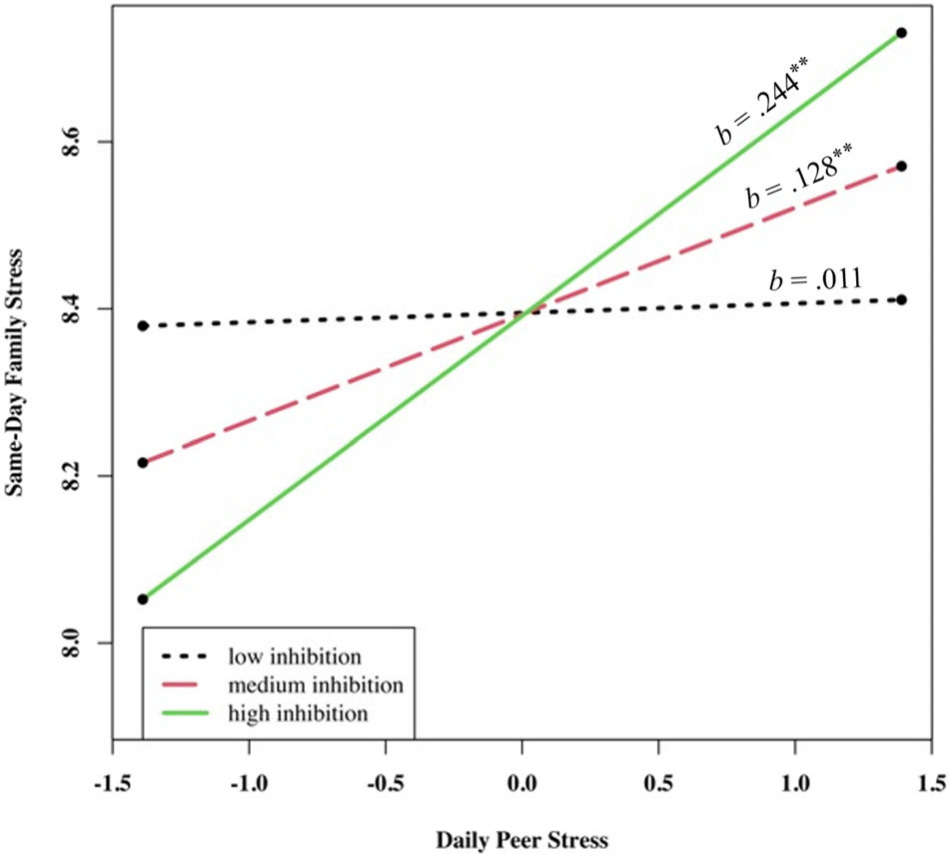
Interaction of Peer Stress and Emotional Inhibition in Predicting Same-Day Family Stress. *Note*. ** *p* < 0.01

As shown in Table 2, after controlling for week and day of week, peer stress experienced on a given day was positively associated with same-day family stress (*γ*_*10*_ = 0.13, *p* = 0.010 for the model with emotional inhibition as the moderator). Further, adolescent emotional inhibition significantly moderated the effect of peer stress on same-day family stress (*γ*_*11*_ = 0.29, *p* = 0.006). Follow-up simple slope analyses (Preacher et al., 2006) revealed that for adolescents with medium (*b* = 0.13, *p* = 0.010) and high levels (*b* = 0.24, *p* = 0.002) of emotional inhibition (i.e., the mean and one standard deviation above the mean), there was a positive effect of daily peer stress on same-day family stress (Fig. 1). However, for adolescents with low levels of emotional inhibition (i.e., one standard deviation below the mean), there was no significant association between same-day peer and family stress (*b* = 0.01, *p* = 0.814). Adolescent dysregulation did not moderate the relation between same-day peer and family stress.

**Table 2:** Multilevel Models Predicting Family Stress from Same-Day Peer Stress

	Inhibition		Dysregulation	
Fixed effect	coefficient (*SE*)	*t* ratio	coefficient (*SE*)	*t* ratio
Same-day family stress intercept, *β*_*0*_				
Intercept, *γ*_*00*_	8.39 (0.26)	32.64^***^	9.40 (0.26)	32.60^***^
Average peer stress, *γ*_*01*_	0.74 (0.13)	5.83^***^	0.67 (0.13)	4.94^***^
Moderator, *γ*_*02*_	−0.01 (0.37)	−0.01	0.77 (0.42)	1.84
Same-day peer stress, *β*_*1*_				
Intercept, *γ*_*10*_	0.13 (0.05)	2.59^*^	0.07 (0.05)	1.42
Moderator, *γ*_*11*_	0.28 (0.10)	2.54^**^	0.15 (0.10)	1.51
Week, *β*_*2*_				
Intercept, *γ*_*20*_	−0.41 (0.11)	−3.69^***^	−0.42 (0.11)	−3.73^***^
Day of the week, *β*_*3*_				
Intercept, *γ*_*30*_	−0.14 (0.04)	−3.47^***^	−0.14 (0.04)	−3.47^***^

* *p* < 0.05, ** *p* < 0.01, *** *p* < 0.001

### Model 2: Next-Day Peer-to-Family Stress Spillover

**Fig. 2:**
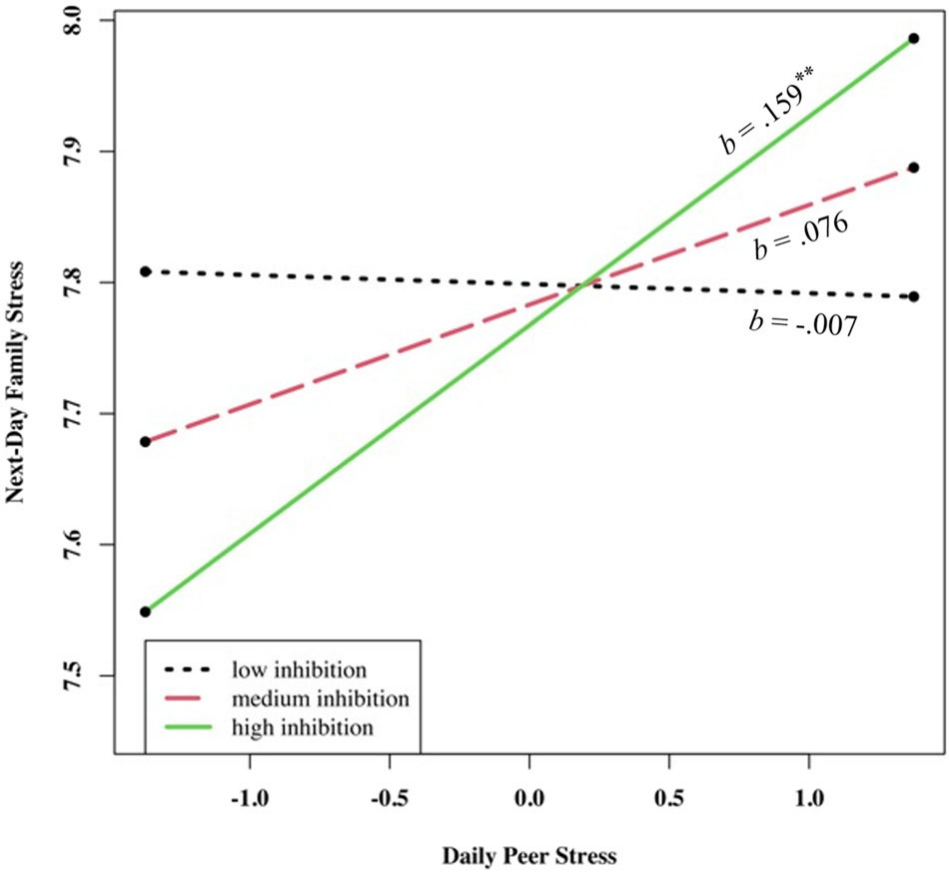
Interaction of Peer Stress and Emotional Inhibition in Predicting Next-Day Family Stress. *Note*. ** *p* < 0.01

There was no significant association between peer stress and next-day family stress. However, there was a significant cross-level interaction between adolescent emotional inhibition and daily peer stress in predicting next-day family stress (*γ*_*11*_ = 0.21, *p* = 0.032). At high levels of adolescent emotional inhibition, peer stress was related to greater next-day family stress (*b* = 0.16, *p* = 0.007). There was no association between peer stress and next-day family stress at medium (*b* = 0.08, *p* = 0.092) or low levels (*b* = −0.01, *p* = 0.908) of adolescent emotional inhibition (see Fig. 2). Adolescent dysregulation did not moderate the association between peer stress and next-day family stress. See Table 3 for all effects.

**Table 3:** Multilevel Models Predicting Next-Day Family Stress from Peer Stress

	Inhibition		Dysregulation	
Fixed effect	coefficient (*SE*)	*t* ratio	coefficient (*SE*)	*t* ratio
Next-day family stress intercept, *β*_*0*_				
Intercept, *γ*_*00*_	7.78 (0.29)	26.53^***^	7.80 (0.30)	26.39^***^
Average Peer Stress, *γ*_*01*_	0.77 (0.12)	6.25^***^	0.69 (0.13)	5.20^***^
Moderator, *γ*_*02*_	−0.04 (0.37)	−0.10	0.78 (0.44)	1.75
Same-day peer stress, *β*_*1*_				
Intercept, *γ*_*10*_	0.08 (0.05)	1.69	0.03 (0.06)	0.50
Moderator, *γ*_*11*_	0.21 (0.10)	2.15^*^	0.10 (0.10)	1.05
Week, *β*_*2*_				
Intercept, *γ*_*20*_	−0.31 (0.13)	−2.42^*^	−0.32 (0.13)	−2.46^*^
Day of the week, *β*_*3*_				
Intercept, *γ*_*30*_	−0.02 (0.05)	−0.53	−0.03 (0.05)	−0.56
Same-day family stress, *β*_*4*_				
Intercept, *γ*_*40*_	−0.10 (0.03)	−4.00^***^	−0.10 (0.03)	−4.00^***^

* *p* < 0.05, *** *p* < 0.001

### Model 3: Next-Day Family-to-Peer Stress Spillover

Family stress at home was not significantly associated with next-day peer stress (for the model with emotional inhibition as the moderator, *γ*_*00*_ = 0.03, *p* = 0.085; for the model with dysregulation as the moderator, *γ*_*00*_ = 0.04, *p* = 0.074). Further, neither emotional inhibition (*γ*_*11*_ = −0.07, *p* = 0.141) nor dysregulation (*γ*_*11*_ = 0.01, *p* = 0.887) moderated this association (see Table 4).

**Table 4:** Multilevel Models Predicting Next-Day Peer Stress from Family Stress

	Inhibition		Dysregulation	
Fixed effect	Coefficient (*SE*)	*t* ratio	Coefficient (*SE*)	*t* ratio
Next-day peer stress intercept, *β*_*0*_				
Intercept, *γ*_*00*_	4.79 (0.16)	29.58^***^	4.79 (0.16)	29.64^***^
Average Family Stress, *γ*_*01*_	0.21 (0.06)	3.53^***^	0.18 (0.06)	3.23^***^
Moderator, *γ*_*02*_	−0.08 (0.17)	−0.49	0.92 (0.20)	4.62^***^
Same-day family stress, *β*_*1*_				
Intercept, *γ*_*10*_	0.03 (0.03)	1.73	0.04 (0.02)	1.79
Moderator, *γ*_*11*_	−0.07 (0.05)	−1.48	0.01 (0.04)	0.14
Week, *β*_*2*_				
Intercept, *γ*_*20*_	−0.04 (0.08)	−0.44	−0.04 (0.08)	−0.46
Day of the week, *β*_*3*_				
Intercept, *γ*_*30*_	−0.03 (0.03)	−1.02	−0.03 (0.03)	−1.01
Same-day peer stress, *β*_*4*_				
Intercept, *γ*_*40*_	−0.12 (0.03)	−4.13^***^	−0.11 (0.03)	−3.93^***^

*** *p* < 0.001

### Sensitivity Analyses

The models assessing next-day effects were reanalyzed. Observations from the 6th day were excluded when used as the outcome variable to ensure that the results were not influenced by the transition across the weekend. These analyses yielded similar outcomes. Adolescent inhibition was found to moderate the association between daily peer stress and next-day family stress (*γ*_*11*_ = 0.29, *p* = 0.003). Additionally, daily family stress predicted next-day peer stress (*γ*_*10*_ = 0.04, *p* = 0.038 in the model with dysregulation as the moderator). No other relationships were significant, as detailed in the Supplementary Results Tables S1–S2.

To examine the potential impacts of family demographics, adolescent age and gender, parent age and gender, parental educational level, monthly household income, and ethcinity were included as level 2 predictors. Consistent findings emerged. Adolescent inhibition was found to moderate the association between daily peer stress and same-day family stress (*γ*_*18*_ = 0.28, *p* = 0.014). Moreover, adolescent inhibition moderated the association between daily peer stress and next-day family stress (*γ*_*18*_ = 0.21, *p* = 0.025). No other significant relationships were observed (Supplementary Results Tables S3–S5).

## Discussion

Bioecological theory suggests that the contexts in which adolescents exist are interconnected and thus, are mutually influential (Bronfenbrenner & Ceci, 1994). Emerging evidence supports a reciprocal relationship between daily peer stress at school and parent-adolescent interactions at home, highlighting a process of daily stress spillover (Bai et al., 2017; Mayfield & Fosco, 2021). However, the temporal sequence of these spillover effects remains unclear, with few studies exploring how variations in emotion regulation difficulties might influence this daily process. This study advances the existing literature by using a study design that allows for an assessment of temporal precedence, examining the potential impact of emotion regulation difficulties on the spillover process, and applying a cultural lens to study questions with a sample of Chinese adolescents. It was expected that daily stress from peers and family stress would have a spillover effect on each other. It was also hypothesized that this spillover process would be greatest in the context of emotion regulation difficulties. Study hypotheses were partially supported.

The hypothesis that same-day peer stress at school would spillover to family stress later at home was partially supported. Specifically, on days when adolescents experienced more peer problems, such as arguing with friends at school, these peer stressors persisted beyond the school day and negatively influenced adolescents’ later interactions with parents at home. The finding of same-day peer stress spillover is in line with previous research on Western adolescents, which has demonstrated a significant association between same-day peer and family stress (Flook & Fuligni, 2008; Timmons & Margolin, 2015). Additionaly, this study extends the understanding of the daily stress spillover process with Chinese adolescents. This extension of prior work is particularly relevant given the extended time Chinese adolescents spend in the school context (Xu & Minca, 2008). Further, Chinese culture places a great emphasis on interpersonal harmony, with cultural norms and values centered around group cohesion (Oyserman et al., 2002). In the context of these cultural values, Chinese adolescents likely place significant emphasis on maintaining friendships, perhaps making peer stress, when it does occur, particularly distressing and thus explaining the spillover of peer stress to the family context.

Contrary to the hypothesis, the main effect of peer stress on next-day family stress was not significant, suggesting that peer stress did not directly spillover to next-day family stress. This finding, though unexpected, is consistent with the idea that the effect of same-day stress spillover is more pronounced than that of lagged associations (Kiang & Buchanan, 2014). Since parent-adolescent relationships are dynamic and subject to daily fluctuations, they may be more susceptible to immediate influences, such as peer interactions within the same day. As a result, the influence of peer stress on family dynamics may tend to diminish over time and generally not extend beyond the day it occurs.

Importantly, however, emotional inhibition moderated both same- and next-day peer-to-family stress spillover associations. For adolescents with higher emotional inhibition, daily peer stress positively predicted same- and next-day family stress. By contrast, for adolescents with lower reported use of emotional inhibition, no same- or next-day associations between peer and family stress were detected. In other words, Chinese adolescents with higher emotional inhibition were highly vulnerable to same- and next-day peer-to-family stress spillover.

These moderation findings are congruent with previous findings from Western samples (Cameron & Overall, 2018; Srivastava et al., 2009). In Western society, emotional inhibition is typically related to reduced relationship satisfaction and greater reported interpersonal disturbances compared to a lack of emotional inhibition (Cameron & Overall, 2018; Gross & John, 2003). Our results indicate that, for Chinese adolescents, there may be a similar cost to the utilization of emotional inhibition as a technique for managing interpersonal stress. Consistent with theory, emotional inhibition may alter the outward expression of negative emotions but not the internal experience of negative emotions. This emotion regulation strategy may leave adolescents with residual negative emotions despite the lack of outward appearance of distress (Brockman et al., 2017; Gross, 2014). An alternative explanation is that external emotional expression, rather than internal emotional inhibition, is a mechanism through which individuals signal others that they need support. Inhibiting emotions may lead adolescents to diminish the external support they receive from others, exacerbating experienced negative emotions and minimizing support in coping with stress (Chervonsky & Hunt, 2017; Schacter & Margolin, 2019). Without being addressed, adolescents’ peer stress likely persists from one day to the next (Chung et al., 2011; Lehman & Repetti, 2007). Thus, emotion inhibition may facilitate broader group harmony in the Chinese context, but it can also exacerbate stress management problems in adolescents.

In contrast to hypotheses, family stress did not significantly predict next-day peer stress, nor was this effect moderated by difficulties with emotion regulation. This result deviates from previous studies involving Western adolescents, which have indicated that family conflict can impact peer interactions the next day (Chung et al., 2011; Timmons & Margolin, 2015). This discrepancy might be attributed to the fact that Chinese adolescents spend considerable time with their peers (Lam et al., 2014). They spend most of their waking hours in the school environment as their school hours begin before 8 AM and end after 5 PM. Extended time at school provides greater opportunities for adolescents to both engage with peers and have potentially more conflictual interactions with their classmates (Xu & Minca, 2008). Further, adolescents often place a great emphasis on both peer relationships and exercising independence from their families more than ever before in development (Hale & Zeman, 2023). Once at home from school, Chinese adolescents likely spend no more than a few hours engaging with their parents prior to going to sleep, perhaps minimizing the impact of familial stress on next-day peer stress. Our assessment of family stress was also collected just prior to adolescents going to sleep (~9 PM), giving adolescents approximately 20 h in between their reporting of family stress and next-day reported peer stress. In this 20-h window, adolescents partook in an entire school day, potentially distracting them from the prior evening’s familial stress and thus helping to explain the lack of effects.

Of interest, emotion dysregulation did not moderate the same- or next-day association between peer and family stress, contrary to previous studies in other cultural groups (e.g., Adrian et al., 2019; Suveg et al., 2014). These lack of findings may be because, in Chinese culture, dysregulation is not a common strategy for regulating emotions. Rather, Chinese culture places a high value on emotional restraint and inhibition (Wei et al., 2013), whereas excessive emotional expression is evaluated negatively and thought to result in disharmonious interpersonal relationships (Deng et al., 2019). Thus, Chinese adolescents may be less inclined to employ a counter-cultural emotion regulation strategy such as dysregulation as a way of coping with intense negative emotions. Indeed, examination of mean values suggests that Chinese adolescents used inhibition more than they did dysregulation. Additionally, while dysregulation is thought to be a maladaptive emotion regulation strategy, it may not result in intense, residual negative emotions, unlike emotional inhibition (e.g., Aldao et al., 2010; Brockman et al., 2017). Rather, dysregulation may be an outward release of experienced negative emotions. Consequently, the stress an adolescent experiences in one social context might not carry over into another domain as adolescents are not continually attempting to suppress their own negative emotions.

### Limitations and Future Directions

This study employed a rigorous methodological and statistical approach to examine stress spillover between peer- and family- contexts within a cultural framework. Despite these strengths, limitations are noted. First, although the directionality of spillover can be established using multiple assessments during the day, we cannot determine causality. That is, although we were able to draw conclusions about the temporal sequence of events, we cannot infer whether peer stress always preceded reported family stress. Second, daily diary entries on stress were based on self-reports from adolescents. Relying exclusively on self-report introduces the possibility of shared method variance, which could potential inflate the results. Future studies incorporating multiple informants may be helpful in addressing this current limitation. Third, while a strength of this study includes the novel investigation of stress spillover in Chinese adolescents, the present sample was relatively homogenous regarding socioeconomic backgrounds. Future work could engage individuals from diverse sociodemographic backgrounds. Fourth, the current study assessed the frequency at which adolescents use emotional inhibition and dysregulation as a general response to negative emotions, without specifically considering peer and family stress experiences. Future research should consider the daily assessment of adolescent emotion regulation difficulties and how these challenges relate to stress spillover to gain a more comprehensive understanding.

Finally, this study exclusively examined the spillover of stress between family and school contexts, neglecting the assessment of the positive dimensions of parent-adolescent relationships (e.g., supportive interactions and positive communication). These positive aspects are pivotal in comprehensively understanding the spillover process and the moderating influence of emotion regulation within it. For instance, perceived high levels of parental support and positive family communication may encourage adolescents to more freely express their stress and negative emotions. This openness, in turn, may reduce their reliance on emotional inhibition as a strategy for emotion regulation, thereby alleviating the school-to-family stress spillover effect (Bryant & DeMorris, 2016). Future research could include assessments of parent-adolescent relationships to enhance an understanding of the spillover process.

## Conclusion

Reciprocal relationships between peer and family stress have been observed in adolescents’ daily lives, revealing a stress spillover effect within school and family contexts. However, simultaneous assessment of these daily stressors offers limited insight into their sequential impacts across different social contexts. Moreover, the role of adolescents’ emotion regulation abilities in moderating this spillover is underexplored, with existing studies largely neglecting non-Western adolescents and the potential impact of cultural variations on emotion regulation and stress dynamics. The current study examined same- and next-day effects of peer and family stress on one another as well as the moderating role of emotion regulation difficulties among Chinese adolescents. Daily peer stress was positively associated with same-day family stress at home. However, neither peer- nor family stress was associated with one another the following day. Emotional inhibition impacted the stress spillover process—adolescents with greater emotional inhibition experienced both same- and next-day peer-to-family stress spillover. Findings from this study contribute to existing literature by elucidating the temporal sequence of daily peer-to-family stress spillover and suggest that emotional inhibition, while culturally adaptive to maintain interpersonal harmony, may be maladaptive for stress management for Chinese adolescents.

### Supplementary Information

Supplemental Material
