# Supplementary material for: Peer Stress Spills Over to Family Stress in the Context of Emotion Regulation Difficulties: A Daily Diary Study with Chinese Adolescents
Source: J Youth Adolesc. 2024 Mar 11;53(6):1415–27. doi: 10.1007/s10964-024-01962-3 (PMC11045594; doi:10.1007/s10964-024-01962-3)
Supplement: Supplementary file 1 — Supplemental Material [file 10964_2024_1962_MOESM1_ESM.docx]

**Online Supplementary Materials for:**

**Peer Stress Spills Over to Family Stress in the Context of Emotion Regulation Difficulties: A Daily Diary Study with Chinese Adolescents**

**Table S1**

*Multilevel Models Predicting Next-Day Family Stress from Peer Stress, Excluding Observations from the 6^th^ Day*

|  | Inhibition | |  | Dysregulation | |
| --- | --- | --- | --- | --- | --- |
| Fixed effect | coefficient (*SE*) | *t* ratio |  | coefficient (*SE*) | *t* ratio |
| Next-day family stress intercept, *β_0_* |  |  |  |  |  |
| Intercept, *γ_00_* | 7.78 (.30) | 25.87^***^ |  | 7.80 (.30) | 25.94^***^ |
| Average Peer Stress, *γ_01_* | .77 (.12) | 6.39^***^ |  | .68 (.13) | 5.09^***^ |
| Moderator, *γ_02_* | -.04 (.37) | .12 |  | .86 (.45) | 1.90 |
| Same-day peer stress, *β_1_* |  |  |  |  |  |
| Intercept, *γ_10_* | .11 (.05) | 2.21 |  | .04 (.05) | .73 |
| Moderator, *γ_11_* | **.29 (.10)** | **2.97^*^** |  | .19 (.10) | 1.95 |
| Week, *β_2_* |  |  |  |  |  |
| Intercept, *γ_20_* | -.33 (.13) | -2.57^*^ |  | -.34 (.13) | -2.63^**^ |
| Day of the week, *β_3_* |  |  |  |  |  |
| Intercept, *γ_30_* | -.01 (.06) | -.13 |  | -.01 (.06) | -.15 |
| Same-day family stress, *β_4_* |  |  |  |  |  |
| Intercept, *γ_40_* | -.09 (.03) | -3.06^**^ |  | -.09 (.03) | -3.03^**^ |

*Note.* * *p* < .05, ** *p* < .01, *** *p* < .001.

**Table S2**

*Multilevel Models Predicting Next-Day Peer Stress from Family Stress, Excluding Observations from the 6^th^ Day*

|  | Inhibition | |  | Dysregulation | |
| --- | --- | --- | --- | --- | --- |
| Fixed effect | Coefficient (*SE*) | *t* ratio |  | Coefficient (*SE*) | *t* ratio |
| Next-day peer stress intercept, *β_0_* |  |  |  |  |  |
| Intercept, *γ_00_* | 4.74 (.15) | 31.13^***^ |  | 4.73 (.15) | 31.02^***^ |
| Average Family Stress, *γ_01_* | .19 (.06) | 3.39^***^ |  | .17 (.05) | 3.02^**^ |
| Moderator, *γ_02_* | -.07 (.16) | -.43 |  | .96 (.20) | 4.81^***^ |
| Same-day family stress, *β_1_* |  |  |  |  |  |
| Intercept, *γ_10_* | .04 (.02) | 1.91 |  | .04 (.02) | 2.08^*^ |
| Moderator, *γ_11_* | -.09 (.05) | -1.82 |  | -.02 (.05) | -.46 |
| Week, *β_2_* |  |  |  |  |  |
| Intercept, *γ_20_* | -.04 (.08) | -.54 |  | -.04 (.08) | -.54 |
| Day of the week, *β_3_* |  |  |  |  |  |
| Intercept, *γ_30_* | .01 (.03) | .02 |  | .02 (.03) | .08 |
| Same-day peer stress, *β_4_* |  |  |  |  |  |
| Intercept, *γ_40_* | -.09 (.03) | -2.86^**^ |  | -.09 (.03) | -2.75^**^ |

*Note.* * *p* < .05, ** *p* < .01, *** *p* < .001.

**Table S3**

*Multilevel Models Predicting Family Stress from Same-Day Peer Stress with All Control Variables*

|  | Inhibition | |  | Dysregulation | |
| --- | --- | --- | --- | --- | --- |
| Fixed effect | coefficient (*SE*) | *t* ratio |  | coefficient (*SE*) | *t* ratio |
| Same-day family stress intercept, *β_0_* |  |  |  |  |  |
| Intercept, *γ_00_* | 8.23 (.41) | 21.32^***^ |  | 8.74 (.41) | 21.39^***^ |
| Average peer stress, *γ_01_* | .75 (.13) | 5.82^***^ |  | .68 (.14) | 4.94^***^ |
| Child age, *γ_02_* | -.22 (.17) | -1.28 |  | -.19 (.17) | -1.13 |
| Child gender, *γ_03_* | -.36 (.30) | -1.22 |  | -.38 (.30) | -1.27 |
| Parent age, *γ_04_* | -.01 (.02) | -.02 |  | -.01 (.02) | -.04 |
| Parent gender, *γ_05_* | -.20 (.38) | -.53 |  | -.19 (.38) | -.50 |
| Parent educational level, *γ_06_* | -.14 (.16) | -.92 |  | -.15 (.16) | -.96 |
| Household income, *γ_07_* | .01 (.07) | .06 |  | .01 (.07) | .06 |
| Ethnicity, *γ_08_* | -.06 (.27) | -.21 |  | -.09 (.26) | -.35 |
| Moderator, *γ_09_* | -.01 (.36) | -.01 |  | .76 (.42) | 1.81 |
| Same-day peer stress, *β_1_* |  |  |  |  |  |
| Intercept, *γ_10_* | .12 (.12) | .10 |  | .03 (.12) | .22 |
| Child age, *γ_11_* | .03 (.06) | .40 |  | .02 (.06) | .40 |
| Child gender, *γ_12_* | .01 (.10) | .05 |  | .02 (.10) | .25 |
| Parent age, *γ_13_* | .01 (.01) | .67 |  | .01 (.01) | .61 |
| Parent gender, *γ_14_* | .01 (.13) | .02 |  | .03 (.13) | .25 |
| Parent educational level, *γ_15_* | .08 (.05) | 1.42 |  | .09 (.05) | 1.77 |
| Household income, *γ_16_* | -.01 (.02) | -.18 |  | -.01 (.02) | -.01 |
| Ethnicity, *γ_17_* | .09 (.14) | .65 |  | .14 (.15) | .98 |
| Moderator, *γ_18_* | **.28 (.12)** | **2.47^*^** |  | .14 (.11) | 1.37 |
| Week, *β_2_* |  |  |  |  |  |
| Intercept, *γ_20_* | -.42 (.11) | -3.72^***^ |  | -.42 (.11) | -3.77^***^ |
| Day of the week, *β_3_* |  |  |  |  |  |
| Intercept, *γ_30_* | -.14 (.04) | -3.49^***^ |  | -.14 (.04) | -3.50^***^ |

*Note.* * *p* < .05, *** *p* < .001.

**Table S4**

*Multilevel Models Predicting Next-Day Family Stress from Peer Stress with All Control Variables*

|  | Inhibition | |  | Dysregulation | |
| --- | --- | --- | --- | --- | --- |
| Fixed effect | coefficient (*SE*) | *t* ratio |  | coefficient (*SE*) | *t* ratio |
| Next-day family stress intercept, *β_0_* |  |  |  |  |  |
| Intercept, *γ_00_* | 8.12 (.46) | 17.81^***^ |  | 8.12 (.46) | 17.81^***^ |
| Average Peer Stress, *γ_01_* | .79 (.13) | 6.22^***^ |  | .71 (.14) | 5.20^***^ |
| Child age, *γ_02_* | -.22 (.18) | -1.24 |  | -.20 (.18) | -1.11 |
| Child gender, *γ_03_* | -.31 (.31) | -.99 |  | -.32 (.31) | -1.05 |
| Parent age, *γ_04_* | -.01 (.02) | -.25 |  | -.01 (.02) | -.26 |
| Parent gender, *γ_05_* | -.24 (.40) | -.61 |  | -.22 (.40) | -.56 |
| Parent educational level, *γ_06_* | -.16 (.17) | -.95 |  | -.16 (.16) | -.98 |
| Household income, *γ_07_* | .03 (.08) | .34 |  | .03 (.08) | .35 |
| Ethnicity, *γ_08_* | -.10 (.26) | -.37 |  | -.13 (.25) | -.54 |
| Moderator, *γ_09_* | -.02 (.36) | -.06 |  | .77 (.44) | 1.72 |
| Same-day peer stress, *β_1_* |  |  |  |  |  |
| Intercept, *γ_10_* | .14 (.11) | 1.27 |  | .06 (.11) | .52 |
| Child age, *γ_11_* | -.09 (.06) | -1.43 |  | -.06 (.07) | -.96 |
| Child gender, *γ_12_* | .12 (.09) | 1.26 |  | .15 (.09) | 1.60 |
| Parent age, *γ_13_* | -.01 (.01) | -.53 |  | -.01 (.01) | -.47 |
| Parent gender, *γ_14_* | -.15 (.12) | -1.27 |  | -.13 (.12) | -1.05 |
| Parent educational level, *γ_15_* | .01 (.06) | .12 |  | .03 (.06) | .45 |
| Household income, *γ_16_* | -.02 (.02) | -.98 |  | -.02 (.02) | -.86 |
| Ethnicity, *γ_17_* | .04 (.30) | .13 |  | .07 (.30) | .23 |
| Moderator, *γ_18_* | **.21 (.09)** | **2.25^*^** |  | .10 (.10) | .97 |
| Week, *β_2_* |  |  |  |  |  |
| Intercept, *γ_20_* | -.31 (.13) | -2.46^*^ |  | -.32 (.13) | -2.49^*^ |
| Day of the week, *β_3_* |  |  |  |  |  |
| Intercept, *γ_30_* | -.03 (.05) | -.54 |  | -.03 (.05) | -.57 |
| Same-day family stress, *β_4_* |  |  |  |  |  |
| Intercept, *γ_40_* | -.11 (.03) | -4.11^***^ |  | -.10 (.03) | -4.06^***^ |

*Note.* * *p* < .05, *** *p* < .001.

**Table S5**

*Multilevel Models Predicting Next-Day Peer Stress from Family Stress with All Control Variables*

|  | Inhibition | |  | Dysregulation | |
| --- | --- | --- | --- | --- | --- |
| Fixed effect | Coefficient (*SE*) | *t* ratio |  | Coefficient (*SE*) | *t* ratio |
| Next-day peer stress intercept, *β_0_* |  |  |  |  |  |
| Intercept, *γ_00_* | 4.63 (.23) | 20.13^***^ |  | 4.64 (.23) | 20.11^***^ |
| Average Family Stress, *γ_01_* | .21 (.06) | 3.63^***^ |  | .18 (.05) | 3.34^***^ |
| Child age, *γ_02_* | .15 (.11) | 1.31 |  | .16 (.11) | 1.45 |
| Child gender, *γ_03_* | -.05 (.14) | -.37 |  | -.07 (.14) | -.52 |
| Parent age, *γ_04_* | .01 (.01) | .49 |  | .01 (.01) | .40 |
| Parent gender, *γ_05_* | .24 (.16) | 1.53 |  | .25 (.16) | 1.58 |
| Parent educational level, *γ_06_* | .01 (.08) | .08 |  | -.01 (.08) | -.03 |
| Household income, *γ_07_* | -.05 (.04) | -1.27 |  | -.04 (.04) | -1.13 |
| Ethnicity, *γ_08_* | -.05 (.09) | -.55 |  | -.09 (.07) | -1.24 |
| Moderator, *γ_09_* | -.09 (.16) | -.52 |  | .92 (.20) | 4.71^***^ |
| Same-day family stress, *β_1_* |  |  |  |  |  |
| Intercept, *γ_10_* | .01 (.04) | .13 |  | .01 (.04) | .12 |
| Child age, *γ_11_* | .06 (.04) | 1.48 |  | .06 (.04) | 1.52 |
| Child gender, *γ_12_* | .04 (.04) | 1.00 |  | .04 (.04) | .89 |
| Parent age, *γ_13_* | .01 (.01) | .85 |  | .01 (.01) | .89 |
| Parent gender, *γ_14_* | -.02 (.02) | .41 |  | .02 (.04) | .55 |
| Parent educational level, *γ_15_* | .01 (.01) | -1.21 |  | -.02 (.02) | -1.38 |
| Household income, *γ_16_* | -.01 (.03) | .74 |  | .01 (.01) | .79 |
| Ethnicity, *γ_17_* | -.01 (.03) | -.31 |  | -.01 (.03) | -.44 |
| Moderator, *γ_18_* | -.06 (.05) | -1.39 |  | .01 (.04) | .21 |
| Week, *β_2_* |  |  |  |  |  |
| Intercept, *γ_20_* | -.03 (.08) | -.42 |  | -.04 (.08) | -.45 |
| Day of the week, *β_3_* |  |  |  |  |  |
| Intercept, *γ_30_* | -.03 (.03) | -1.01 |  | -.03 (.03) | -1.01 |
| Same-day peer stress, *β_4_* |  |  |  |  |  |
| Intercept, *γ_40_* | -.12 (.03) | -4.09^***^ |  | -.11 (.03) | -3.89^***^ |

*Note.* *** *p* < .001.
